# Adherence to national malaria clinical management and testing guidelines in selected private clinics of Gambela Town, Gambela Region, Ethiopia: a mixed method study

**DOI:** 10.1186/s12936-022-04206-6

**Published:** 2022-06-03

**Authors:** Yamlak Gindola, Desalegn Getahun, David Sugerman, Eric Tongren, Ryan Tokarz, Mesfin Wossen, Kassahun Demissie, Etsehiwot Zemelak, Akuma Okugn, Jimmawork Wendimu, Girmay Hailu, Mesfin Tegistu, Dumesa Begna

**Affiliations:** 1 Ethiopian Public Health Institute, Addis Ababa, Ethiopia; 2 Centers for Disease Control and Prevention, Atlanta, USA; 3 Gambela Regional Health Bureau, Gambela, Gambela Regional Ethiopia; 4 Department of Public Health, St’Paul Hospital Millennium Medical College, Addis Ababa, Ethiopia; 5 Federal Ministry of Health, Addis Ababa, Ethiopia

**Keywords:** Adherence, Malaria, Gambela Town, Ethiopia, Disease guidelines, Private clinics

## Abstract

**Background:**

The World Health Organization World Malaria Report of 2019 indicated an estimated 228 million cases of malaria occurred worldwide in 2018. More than 75% of the total area of Ethiopia is malarious, making malaria a leading public health problem in Ethiopia. Adherence to clinical guidelines improves the quality of care received by patients, thus improving patient outcomes. This study investigates healthcare workers’ adherence to malaria testing and treatment guidelines in selected private clinics of Gambela Town, Ethiopia.

**Methods:**

A mixed study design involving a retrospective review of 425 patient files and 20 healthcare worker interviews in private clinics was implemented. Data were collected using pre-tested data collection forms. The collected data were then cleaned and entered into statistical software for analysis, with a level of significance set at < 0.05. A qualitative analysis was also conducted using healthcare worker interviews to identify the existing barriers to guideline adherence.

**Results:**

Among the 430 cases of suspected malaria, only 65% were tested for malaria. Of those tested, 75% tested positive and 25% tested negative. The most common co-morbidity in patients treated for malaria was anaemia (30%), followed by gastroenteritis (10%). Patients with co-morbidities were more likely to receive appropriate treatment (p = 0.03) compared to those without co-morbidities. All healthcare workers interviewed were aware of the existence of the malaria treatment guidelines. However, many were not aware of the contents of the guidelines and only 40% had been trained to understand the guidelines. Overall, 85% of the workers claimed to adhere to guidelines, with 15% claiming non-adherence.

**Conclusion:**

The gap between knowledge of the malaria treatment guidelines and their application by healthcare workers remains wide. The level of knowledge of these guidelines was also low. Continuous training, follow-up, supportive supervision, and improved adherence to the malaria guidelines are therefore recommended.

## Background

Malaria is a complex disease that differs in epidemiology and public health impact in different parts of the world. This disease affects 3.4 billion people in 104 countries and territories [1]. According to the World Malaria Report 2020, globally there were an estimated 229 million malaria cases in 2019 in 87 malaria-endemic countries, declining from 238 million cases across 108 countries that were malaria-endemic in 2000. Sub-Saharan African countries bear the largest malaria morbidity burden, with 213 million cases (93%) reported in 2018 [2].

Much of Ethiopia is malaria endemic. The country has made major strides in malaria control over the years, however more than 75% of the total area of Ethiopia remains malarious. As a result, malaria is the leading cause of morbidity and mortality in the country [3]. In Ethiopia, this disease is one of the top 10 causes of morbidity, accounting for 17% of all cases and 8% of health facility admissions in 2018 [4]. Since 2006, the three intervention strategies that have been implemented to control malaria are early diagnosis and prompt treatment, vector control and epidemic prevention and control. To accomplish these intervention strategies, the following key activities were implemented: training of health workers, strengthening community participation to enhance prevention measures such as avoiding malaria breeding areas, use of indoor residual spraying and insecticide-treated nets (ITNs), and expanding malaria diagnosis and treatment at the community level through health extension workers (HEWs) [5].

Within Ethiopia, Gambela is one of the most endemic regions for malaria transmission [6]. Annual rainfall is between 500 and 2000 mm and the mean annual temperature between 31 and 42 °C. These environmental factors, along with an average elevation of 526 m above sea level, contribute to the high vector population and subsequent high prevalence of malaria transmission [7]. Despite active prevention and control activities in Gambela, the disease continues to be the leading cause of outpatient department (OPD) visits in adults and children under the age of five years [5].

To combat the burden of this disease, malaria treatment guidelines have been developed at the national and international levels. The World Health Organization (WHO)’s most recent edition of malaria treatment guidelines was published in 2015 [8]. These guidelines cover the diagnosis and treatment of uncomplicated and severe malaria. Ethiopia’s Federal Ministry of Health first published its national guidelines for diagnosis, treatment and prevention of malaria in 2002 [9]. These guidelines have been updated periodically to account for the emergence of new evidence and information gathered through continuous monitoring and evaluation.

The Ethiopian Ministry of Health published its current malaria treatment guidelines in November 2017 [10]. The guidelines aim to improve malaria case management by all healthcare workers to reduce morbidity and mortality, and sets the target of 100% access to effective and affordable malaria treatment. This requires improving the diagnosis of malaria cases using microscopy or multi-species rapid diagnostic tests (RDTs) and providing prompt and effective malaria case management at all health facilities. Malaria diagnosis and treatment are essential components of anti-malaria interventions in the country. Malaria diagnosis consists of a patient’s clinical assessment, microscopic examination of blood slides, and use of multi-species RDT in accordance with the level of the health facility.

In Ethiopia 80% of primary health care is public, while 20% is private [11]. Malaria spending is estimated to cost Ethiopia’s public sector about $200 million annually or 10% of its total health expenditure, while it costs $6.58 million in the private sector for malaria treatment and diagnosis. The Ethiopian private sector is relatively small and fragmented compared to other countries in the region [12]. The availability of high-quality, inexpensive RDTs in the public sector has significantly improved and expanded diagnostic testing. However, in the private sector, RDTs are either non-existent or more expensive than artemisinin-based combination therapy (ACT) [13].

The recommended treatment for uncomplicated malaria is artemether-lumefantrine (AL) and primaquine (for *Plasmodium falciparum*). If an RDT or microscopy reveals a *Plasmodium vivax* infection only, then chloroquine treatment should be given. For complicated (severe) malaria the preferred drugs are: Intravenous (IV) or Intramuscular (IM) artesunate or IM artemether or, IV quinine infusion (if artesunate or artemether are not available). Antimalarial treatments are effective in controlling the disease [14], however, as evidenced by a study in Kenya, one of the continuing problems is therapeutic failure. This results from several factors, including non-adherence to the standard malaria management protocol [15]. A study from nearby Uganda suggests that knowledge about the disease and its treatment protocol is one of the service provider-related factors in non-adherence [16]. Several additional factors can contribute to less-than-optimal adherence by healthcare workers. These factors are related to healthcare worker attitude towards the guidelines, institutional factors such as availability of resource required for guideline implementation, and factors related to the nature of the guidelines, which includes ease of understanding [17, 18]. Ethiopia has not previously conducted studies to assess service provider adherence to malaria treatment guidelines. This study aims to evaluate healthcare worker adherence to national clinical testing and management guidelines for malaria in private clinics in the endemic area of Gambela Town, Gambela Region.

## Methods

**Fig. 1:**
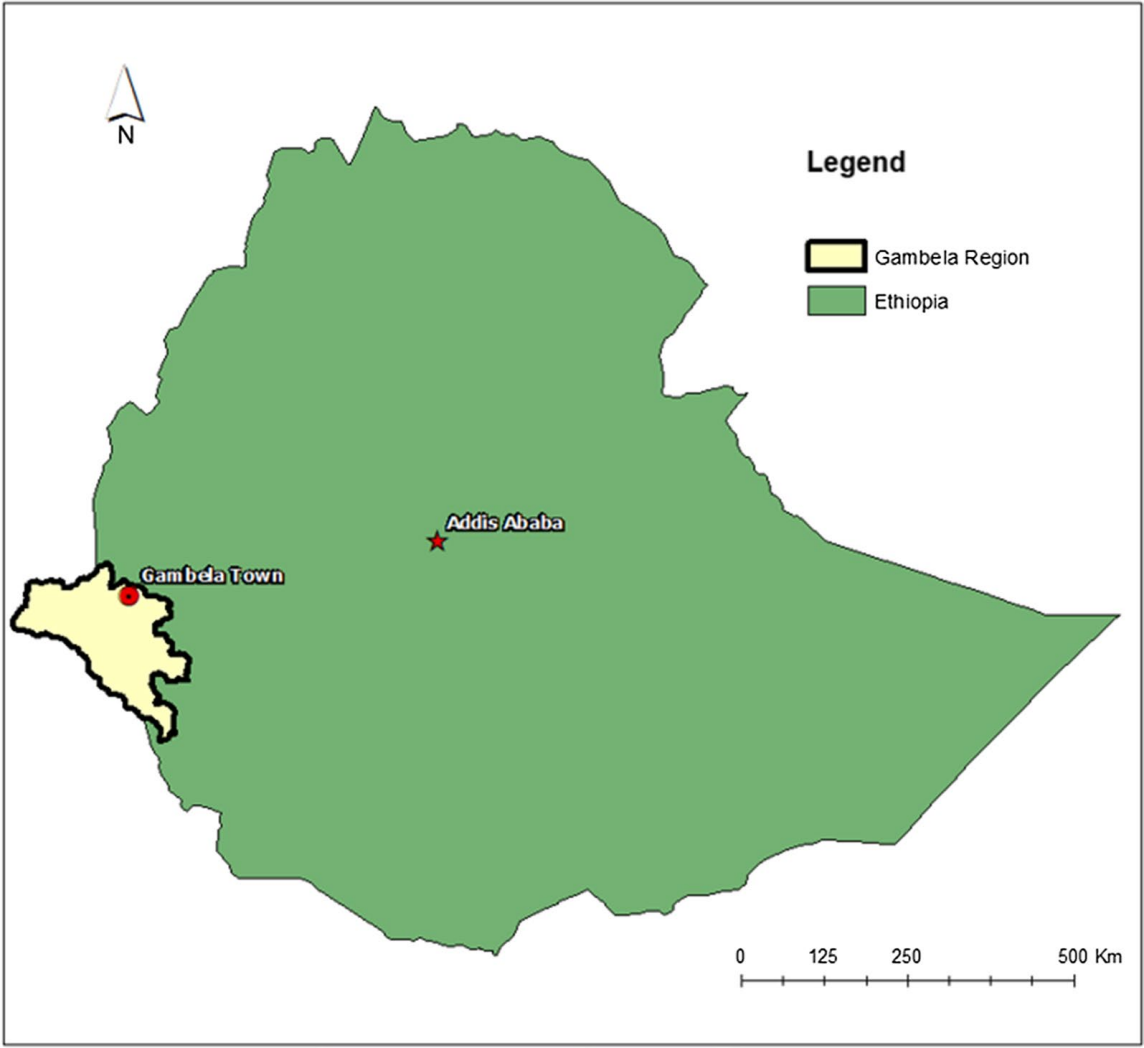
Map of Gambela region

The study was conducted between March and April, 2020 in Gambela Town, the capital of Gambela region (Fig. 1). The town is one of the most endemic parts of the region, demonstrating high malaria transmission throughout the year. A simple random sampling method was used to select private clinics in an area of interest for the president’s malaria initiative (PMI). For the quantitative component of the study. Probability proportional to size (PPS) technique was used to select sample sizes from each of the 15 private clinics, resulting in a sample size requirement of 425.

The population was composed of patients with suspected and confirmed cases of malaria who presented to the selected private clinics in the months of March-August, 2020. Patient files within this date range were reviewed to identify those containing malaria cases, with a total of 3,800 patient record files identified. A data collection form was used to compile data for the quantitative component. After checking for completeness, data were entered into SPSS Data Analysis Software-Version 24 and analyzed. Bivariate analysis was used to examine the association between variables and patient outcomes, with a probability (*p*) value of < 0.05 indicating significance.

The qualitative component utilized a purposive sampling technique to select the health professionals for study participation. The target sample size was 20 healthcare professionals, all of whom had a direct and indirect involvement in diagnosing and managing malaria cases and had completed a consent form prior to their enrolment. Data were collected via an interviewer-administered questionnaire. Themes were identified from each interview. The themes were reviewed after each interview until point of theme saturation was reached. Themes were considered fully developed at the point in the data collection process where no new or relevant themes emerged. Three interviews with no new or relevant information were considered indicative of theme saturation. Once the entire transcript data were categorized into intervention categories/themes, using an initial information organizer-working template, a review was made in order to ensure that the information was correctly and appropriately categorized. It was also rechecked against the original transcripts to ensure that all the information had been categorized.

A diagnosis of malaria was considered only if detection of antibodies against asexual blood stage malaria parasites was found in peripheral blood by microscopy or the *Plasmodium* antigen was detected by RDT. The malaria diagnosis was further classified as uncomplicated malaria or severe/complicated based on the signs and symptoms of the patient. This classification was based on WHO clinical features of uncomplicated and severe malaria. Ethical approval to conduct the study was obtained from Ethiopian Public Health Institute (EPHI) Ethical Review Board.

## Results

The quantitative component indicated a mean patient age of 11 years, with males being the dominant patient demographic at 57% (Table 1). From the total 428 patients who underwent treatment for malaria, a laboratory test was done for 278 (65%) and not done for the remaining 150 (35%) patients (Table 2). Out of 278 patients who underwent laboratory confirmation, 208 (74.8%) tested positive, while 70 (25.2%) tested negative. Treatment with anti-malarial drugs was given to 99% of the patients who tested positive, 95.8% of those who tested negative, and 100% of the untested patients. Of those who tested positive, 107 (51.4%) were classified as uncomplicated malaria and 101 (48.6%) were classified as severe malaria.

**Table 1:** Demographic characteristics of patients

Characteristic	Frequency	Percentage
*Age (years)*		
0–5	212	50
6–13	121	29
> 13	92	22
*Gender*		
Male	240	57
Female	185	44
*All patients*	425	100

**Table 2:** Patients underwent malaria treatment and their laboratory status

Variable		n	%
Lab test done	Yes	278	65
	No	150	35
Type of test	Microscopy	274	98.2
	RDT	5	1.8
Test result	Positive	208	74.8
	Negative	70	25.2
Type of malaria	Uncomplicated	109	25.3
	Sever	101	23.5

Only one case of uncomplicated malaria was treated according to guideline recommendations while 107 (99.1%) cases were managed contrary to the guidelines. These cases were treated with drugs used for severe malaria, parenteral artesunate or quinine. Of the cases meeting the classification of severe malaria, 95 (95%) received guideline concordant management using parenteral artesunate or quinine followed by oral AL. The remaining five cases (5%) received non-concordant management by drugs other than parenteral artesunate or quinine followed by oral AL.

The most common combination of anti-malarials used was quinine and AL 190 (44.7%), followed by artesunate and AL 184 (43%). Five patients (1%) were treated with both quinine and artesunate (Table 3). Outcomes of patients treated in accordance with guidelines compared to those treated contrary to the guidelines are summarized in Table 4. Of the patients who were diagnosed as per guidelines, 96% were discharged, with death, re-admittance and transferred composing the final 4%. Patients not diagnosed per the guidelines had the following outcomes: discharge (94%), death (2%), re-admission (3%) and transfer (< 1%). No statistically significant association was found between testing for malaria and patient outcomes (p = 0.705).

**Table 3:** Combination of anti-malarial drugs used

First drug	Second drug	Frequency (N)	Percentage (%)
Quinine	AL^*^	190	44.7
Artesunate	AL	184	43.3
AL	None	24	5.6
Quinine	D-P^**^	13	3.1
Artesunate	Quinine	5	1.2
Quinine	Artesunate	3	0.7
Artesunate	Proguanil	3	0.7
Artesunate	D-P	2	0.5
Quinine	None	1	0.2

^*^Artemether lumefantrine

^**^Dihydroartemisinin-piperaquine

**Table 4:** Associations between adherent management and patient outcomes

Adherence		Total		Outcome								
				Discharge		Death		Re-admission		Transfer		P-value
		N	%	N	%	N	%	N	%	N	%	
Diagnostic testing	No	149	35	140	94	3	2	5	3.4	1	0.7	0.705
	Yes	278	65	268	96	3	1.1	6	2.2	1	0.4	
Treatment	No	333	78	318	96	4	1.2	10	3	1	0.3	0.491
	Yes	96	22	92	96	2	2.1	1	1	1	1	

All 20 health professionals interviewed in this study were aware of the national treatment guidelines for malaria in Ethiopia. Of this group, 8 (40%) had attended at least one training session on the current national guidelines for the management of malaria. Analysis for possible associations between the training of health workers and adherence to malaria treatment guidelines revealed no statistically significant association (Table 5). Attitudes of the health workers towards the guidelines did not influence their adherence to guidelines. Healthcare workers who participated in the study had a positive opinion about the usefulness of the malaria treatment guidelines, with only two (10%) indicating that they did not find the guidelines useful in their practice. Of those who found the guidelines useful, three (15%) indicated that the guidelines helped to reduce drug waste and were concerned about wasteful use of anti-malarials:

“*Treatment of unconfirmed cases of malaria puts an unnecessary burden on the pharmacy as there are limited resources allocated to drug procurement.”*

**Table 5:** Relationship between profession, training and attitude on adherence to guidelines

	Adherent		Non-adherent		P-value
	n	%	n	%	
*Profession*					
Pharmacist	2	40	3	60	0.17
Nursing	4	80	1	20	
Lab technician	4	100	0	50	
Health officer	3	100	0	33	
General practitioner	2	100	0	0	
Druggist	1	100	0	0	
*Training*					
Yes	8	89	1	11	0.369
No	8	73	3	27	
*Attitude*					
Agreeable Attitude	8	89	1	11	0.637
Disagreeable Attitude	2	67	1	33	
Neutral Attitude	6	75	2	25	

Two (10%) of the respondents indicated that the guidelines simplified clinical decisions as it provided guidance on patient management and when to refer a patient to another facility. By simplifying clinical decisions, the guidelines allow paramedical personnel to manage malaria cases in resource-limited settings such as dispensaries run by nurses. One of the health workers specifically indicated:

“*When I was working at a dispensary and was the only healthcare provider, I was able to know when to refer patients with severe malaria by referring to the guidelines.”*

Most respondents (70%) believed that adherence to malaria treatment guidelines would improve patient outcomes, while one (5%) believed that they would not. The remaining 25% had no opinion on the matter. Of the participants, 17 (85%) claimed that they follow the guideline recommendation in diagnosis and management of malaria. Only three (15%) indicated their past experience in practice as the reason for not following recommendations. This group believed that drugs other than those recommended in the guidelines were more efficacious in treating malaria and produced better outcomes.

“*In our setting, quinine is superior to artesunate. I have had numerous experiences where patients do not get well until they receive parenteral quinine.”*

One respondent believed in the adequacy of clinical suspicion in the diagnosis of malaria, while three (15%) felt the guidelines were not applicable in their setting. One health worker suggests that a clinician with extensive experience can correctly diagnose malaria on clinical suspicion:

“*With experienced clinicians, clinical symptoms are sufficient to make a diagnosis of malaria. Even if a patient tests negative but has these symptoms, an ant-imalarial should be issued.”*

The healthcare workers interviewed indicated that there were some considerable external barriers in obtaining a confirmatory diagnosis of malaria. Nearly all, 18 (90%), thought that there was availability of the necessary laboratory reagents and equipment for malaria diagnosis, yet only seven (35%) thought that the laboratory was adequately staffed with qualified personnel. Staffing issues were a particular concern:

“*There are very many patients who come to the clinic every day and most of them require testing for malaria. We however have very few laboratory technicians who work long hours and this may compromise the quality of their work. The reagents and equipment are available, but the technicians are few.”*

## Discussion

Young children composed the majority of patients involved in this study. Half of the patients treated for malaria at the facilities were aged 0–5 years, while a total of 78% were between 0–13 years. Proper treatment for this demographic is essential as children, especially those under five years old, are particularly vulnerable to this disease.

Parasitological confirmation is a crucial component of treatment, as the result informs the clinician’s decision to prescribe an anti-malarial. However, studies have indicated that the concordance rate between ‘presumptive’ and ‘actual’ parasitological malaria ranges between 10 and 60% [19]. This shows that presumptive treatment for malaria results in other febrile illnesses being treated as malaria, thus endangering the patient and wasting resources. Therefore, the patients clinically diagnosed and treated for malaria in this study are considered to have been inappropriately managed, as only parasitological confirmed cases should be prescribed anti-malarials.

Of the patients who were tested in this study, 75% had a positive result. Almost all (96%) who tested negative were still treated with anti-malarials, contrary to the guidelines which recommend avoiding prescribing anti-malarials. These patients are therefore classified as receiving inappropriate treatment. This number remains unacceptably high and interventions such as conducting regular supervision, mentorship and training are necessary. In a study conducted in Tanzania, 168 patients presented for treatment at private health facilities, with only 63% being tested for malaria. Of those tested, 30% were positive and 70% were negative. Anti-malarials were then issued to all positive results, 14% of negative results, and 28% of those not tested [20]. The prevalence of positive results was higher in this study than that observed in Tanzania [21]. Overtreatment was also more prevalent in this study, as 96% of negative results and 100% of untested patients received anti-malarials. This may be attributed to poor health worker knowledge of the national malaria guidelines or negligence in guideline adherence.

Although statistically significant associations between treatment adherence and patient outcomes were not found, this does not infer that adherence has no influence on outcomes. Such an association would be difficult to determine statistically without an extremely large sample size due to patient variability. When comparing patient outcomes, it was found 67% of deaths occurred in patients who received inappropriate management. Of the 11 re-admitted patients, 90% occurred in patients managed contrary to the guidelines. This is consistent with a study made in Kenya, which established associations between non-adherent treatment and poor patient outcomes [21].

This study found no significant association between previous training of health workers and adherence to malaria treatment guidelines. This should not be construed as indicating that training and supervision do not lead to better adherence to treatment guidelines by health workers. However, studies on the efficacy of training as a method to improve guideline adherence have yielded conflicting results. Some studies show improvement [22], while others show minimum or no improvement of healthcare worker practice [23, 24]. In this context, wider dissemination of guidelines and extensive training of healthcare workers would be beneficial. Continuous medical education on the guidelines should augment training and update healthcare workers on any changes in the recommendations. In addition, in order to maintain quality of training, regular monitoring with standardized checklist and other monitoring tools along with provision of on-job training or orientation should be maintained.

The Federal Ministry of Health has responsibility to control the health sectors (both public and private) across the country, and has a mandate to give legal license to private health sectors to conduct their business; at the same time, the Ministry is fully mandated to monitor and supervise private health sectors whenever necessary [11]. The Health Minister has a vertical and horizontal structure in the health system. The vertical structure allows two-sided communication with the regional health bureaux, zonal health departments and district health offices to work in collaboration towards the common goal. The horizontal structure allows the Minister to work in coordination and collaboration with other sectors, stakeholders, partners and private health sectors. Using the horizontal structure, the Ministry can influence private health sectors in terms of mentoring, supervision and training. The usual practice is whenever the Government health sector invites the private sectors to training and workshops, it will provide the necessary per diem and transportation costs to participants.

## Conclusion

Malaria management was characterized by relatively poor adherence to diagnosis and treatment guidelines. This is despite the existing scientific information showing that adherence to the guidelines leads to improved patient outcomes and deters the emergence of resistance to anti-malarial treatment. Anti-malarial treatment in patients who test negative and those who are untested is still practiced in Ethiopia and other countries. Strategies need to be developed to address this culture of lack of diagnosis and overtreatment. This study found inappropriate diagnosis and treatment of many positive malaria cases. Addressing these issues would prove beneficial both long and short term for Ethiopia and its at-risk malaria population. Some recommendations that could solve these shortcomings despite the provision of training include enhanced supportive supervision, job aids, internal and external audits and feedback sessions.

## Data Availability

Due to confidentiality agreements, supporting data can only be made available to bona fide researchers subject to a non-disclosure agreement. Details of the data and how to request access are available from *[*www.ephi.gov.et*]* at *[EPHI website]."*
